# If We Offer it, Will They Accept? Factors Affecting Patient Use Intentions of Personal Health Records and Secure Messaging

**DOI:** 10.2196/jmir.2243

**Published:** 2013-02-26

**Authors:** Ritu Agarwal, Catherine Anderson, Jesus Zarate, Claudine Ward

**Affiliations:** ^1^Center for Health Information and Decision SystemsRobert H. Smith School of BusinessUniversity of MarylandCollege Park, MDUnited States; ^2^University of VirginiaSchool of Professional EducationFairfax, VAUnited States; ^3^IMS Government SolutionsChief Information Officer, Practice Leader Healthcare SolutionsFalls Church, VAUnited States; ^4^US Air ForceHealth Promotion (AFMOA/SGHC)Lackland AFB, TXUnited States

**Keywords:** personal health record, technology acceptance, secure messaging, patient-centered care, employer sponsored PHR

## Abstract

**Background:**

Personal health records (PHRs) are an important tool for empowering patients and stimulating health action. To date, the volitional adoption of publicly available PHRs by consumers has been low. This may be partly due to patient concerns about issues such as data security, accuracy of the clinical information stored in the PHR, and challenges with keeping the information updated. One potential solution to mitigate concerns about security, accuracy, and updating of information that may accelerate technology adoption is the provision of PHRs by employers where the PHR is pre-populated with patients’ health data. Increasingly, employers and payers are offering this technology to employees as a mechanism for greater patient engagement in health and well-being.

**Objective:**

Little is known about the antecedents of PHR acceptance in the context of an employer sponsored PHR system. Using social cognitive theory as a lens, we theorized and empirically tested how individual factors (patient activation and provider satisfaction) and two environment factors (technology and organization) influence patient intentions to use a PHR among early adopters of the technology. In technology factors, we studied tool empowerment potential and value of tool functionality. In organization factors, we focused on communication tactics deployed by the organization during PHR rollout.

**Methods:**

We conducted cross-sectional analysis of field data collected during the first 3 months post go-live of the deployment of a PHR with secure messaging implemented by the Air Force Medical Service at Elmendorf Air Force Base in Alaska in December 2010. A questionnaire with validated measures was designed and completed by 283 participants. The research model was estimated using moderated multiple regression.

**Results:**

Provider satisfaction, interactions between environmental factors (communication tactics and value of the tool functionality), and interactions between patient activation and tool empowerment potential were significantly (*P*<.05) associated with behavioral intentions to use the PHR tool. The independent variables collectively explained 42% of the variance in behavioral intentions.

**Conclusions:**

The study demonstrated that individual and environmental factors influence intentions to use the PHR. Patients who were more satisfied with their provider had higher use intentions. For patients who perceived the health care process management support features of the tool to be of significant value, communication tactics served to increase their use intentions. Finally, patients who believed the tool to be empowering demonstrated higher intentions to use, which were further enhanced for highly activated patients. The findings highlight the importance of communication tactics and technology characteristics and have implications for the management of PHR implementations.

## Introduction

### Background

Patient-centered care is a core component of the Institute of Medicine’s quality aims and of the Affordable Care Act of 2011. Policy initiatives for health care transformation envision a health care system that is patient-centric [1], where the patient is a focal and engaged player in managing his/her health and health care. A critical element of this vision is patient empowerment with tools and technologies that support health information management, exchange, and use [2-4]. Personal health records (PHRs) are an important class of health information management tools that enable patients to store, retrieve, and manage their personal health information and ultimately, to stimulate health action [5]. However, while approximately 70 million people in the United States have access to some type of PHR [6] and despite the value potential of PHRs for engaging consumers as active participants in their health and well-being, the volitional uptake of PHRs has been slow. Although adoption rates of PHRs are not widely reported in the literature, studies [6] noted that the adoption of PHRs by patients is generally modest. A consumer survey conducted in 2011 revealed that broad-based consumer adoption of PHRs is not occurring, with only 7% of consumers reporting they had ever used a PHR. Google’s announcement that it plans to close its health records service [7] in 2012 further underscores the limited diffusion of consumer controlled PHRs, a phenomenon not restricted to the United States alone [8].

An alternative to the consumer controlled PHR is one offered by employers as a service to their employees [9]. This mode of PHR delivery addresses one of the critical concerns voiced by consumers in regard to PHRs they adopt on their own, which is, entering and updating personal health information. Additionally, the employer may be in a better position than third parties to alleviate employee’s concerns about security, another significant impediment to PHR use [10,11]. However, within the context of such employer-sponsored PHRs, there is limited research examining various aspects of PHR deployment and acceptance, and many unanswered questions remain [12,13].

The aim of this paper was to understand the factors that influence individuals’ intentions to use a PHR provided by the employer. Our specific focus was on understanding what influences the behavior of early adopters of PHRs, so that PHR adoption can be accelerated. We report findings from the deployment of a PHR implemented by the Air Force Medical Service (AFMS) at Elmendorf Air Force Base in Alaska in December 2010. The PHR tool supported entry and management of health information directly by patients, integrates with the patients’ clinical records, offered access to a wide range of educational materials, and supported secure patient-provider messaging (SM).

Studies of consumer health information technology acceptance have limited their focus to patient demographics and health variables or general perceptions of the technology (eg, ease of use and usefulness) [14-18]. While these studies provide valuable insight into the individual technology adoption process, there is limited understanding of factors driving PHR acceptance in employer-sponsored contexts, especially those factors associated with the deploying organization. Further, there is a paucity of work examining how usage intentions are formed in the initial stage after the adoption decision has been made. We addressed these gaps in knowledge by developing and testing a model that was theoretically grounded and incorporated factors uniquely relevant to the deployment context. The social cognitive theory (SCT) [19] provided the theoretical foundation for the research model. Factors studied included perceptions of the technology, communication tactics deployed by the employer, and individual characteristics of patient activation and satisfaction with their provider. Findings from a detailed survey of 283 early adopters provided insight into patients who were more likely to use the PHR and the actions an organization could proactively take to influence usage intentions.

### Theoretical Foundation and Research Hypotheses

SCT describes individual behavior as mutually dependent upon contextual or environmental factors, and individual factors that reflect the individual’s prior history, skills, and innate propensities. SCT is a robust theory that has been successfully applied to explain phenomena across various domains including behavior towards information technology [20], organizational behavior, training and education, and the psychology underlying individual choices [19,21-25].

**Figure 1:**
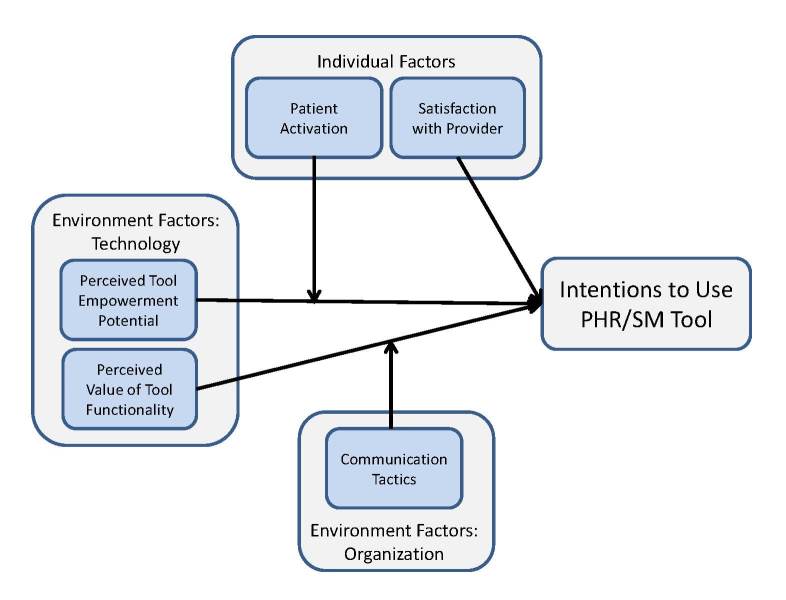
PHR acceptance model.

The PHR acceptance model is depicted in Figure 1. Drawing on SCT, we hypothesized that individual and environmental factors, specifically, technology and organization, will interact to influence acceptance of the tool. We measured individual acceptance of the PHR by self-reported behavioral intention to use the tool—a widely used dependent variable in technology acceptance research [18,26-29] with strong predictive power for actual use behavior [29-31].

### Individual Factors

SCT asserts that an individual’s background, expectations, traits, and skills influence their decisions and behavior [19]. Factors especially salient in the context of PHR adoption are patients’ satisfaction with their health care provider [32-35] and the extent to which patients believe they are in control of their own health care (ie, patient activation) [36-38]. With regard to the patient-provider relationship, the effect of a positive relationship on adherence to recommended treatment regimens is well documented [32-34]. Studies have generally been conducted in the context of chronic disease conditions where frequent interactions with the provider were required and sustained effort was needed of the patient to manage his/her disease (eg, HIV or diabetes) [32-34]. Collectively, this prior work demonstrated that stronger patient-provider relationships could increase positive health-related intentions and behaviors.

Research on technology acceptance and use and the patient-provider relationship has shown the effect of technology use on various aspects of the relationship and not the reverse [39-41]. One exception was observed in a qualitative study of patient focus groups conducted by Zickmund et al [35]. Their findings indicated that interest in using a patient portal was negatively associated with satisfaction with the patient-provider relationship. However, they attributed the limitations of their work to a small sample size, selection bias, and the focus on a single disease (diabetes), and called for more studies on the association between the patient-provider relationship and the use of technology that facilitates health information availability and communication with providers. Drawing upon the stronger evidence in support of a positive association between the patient-provider relationship and positive health action in the more widely studied treatment regimen contexts, and to the degree that a PHR facilitates greater attention to health-related issues, we hypothesized that a positive patient-provider relationship would amplify intentions to use the PHR.

*Hypothesis 1: Satisfaction with the health care provider is positively associated with intentions to use the PHR tool.*

Studies have shown that patients who demonstrated higher levels of knowledge, skill, and confidence in their ability to self-manage their health (ie, they are “activated”) exhibited healthier behaviors including reading about drug interactions, exercising, and eating right [36]. Highly activated patients with chronic conditions were more likely to adhere to prescribed medications, use self-management services (including the use of educational websites), and follow suggested self-management behaviors [36-38]. Patient activation is akin to self-efficacy, which is a central construct in the person component of SCT. Both patient activation and self-efficacy refer to an individual’s perception of their ability to accomplish a particular task, in this case, health self-management. Based on prior findings, we expect that patients with higher levels of activation will be more likely to accept a technology designed to provide them with access to their health information and facilitate interactions with their providers. However, we expect patient activation to moderate one of the environmental factors of interest in this study, therefore we do not hypothesize a main effect of patient activation on behavioral intentions.

### Environment Factors

According to SCT, perceptions concerning the environment, including available technologies and mass media communications, can promote or inhibit relevant behaviors. In the context of this study, PHRs represent a mechanism through which an individual can gain access to their medical record and securely message their provider. We examined 2 factors associated with perceptions of the tool. First, we studied the influence of perceptions about the value of specific functions provided by the tool on use intentions. Second, we examined the influence of a more affective perceptual measure, which captured patients’ beliefs about how the use of the tool might empower them, on behavioral intentions. We also investigated the influence of communication tactics, an organizational factor, on use intentions.

### Technology Factors

The basic form of PHRs typically store medical information and allow users to access, add to, or modify this information [42]. The functionality present in the system implemented by the AFMS at Elmendorf incorporated additional capability to access educational material and securely message providers. Patients who believe that information availability and a new way to communicate with providers affords them greater control over their health care situation, may be more motivated to accept the technology. This effect will be stronger for patients who are already highly knowledgeable about their health status and confident in their ability to self-manage their health. High levels of activation combined with a belief that the PHR tool will result in further empowerment through the increased access to information, enhanced control, and better organization should amplify usage intentions.

*Hypothesis 2: Patient activation will enhance the positive association between tool empowerment potential and intentions to use the PHR tool.*

PHR tools in general can incorporate a wide range of functions that support different tasks and activities, each of which has distinctive instrumental value for patients [42]. For example, the PHR tool examined in this study allowed the patient to access medical information from any Web-enabled computer anytime, track lab results, record immunizations, receive health-specific reminders, and securely message the provider. Research has shown that the perception of tool value is a strong driver of technology acceptance [29]. Therefore, we expect that patients who find the PHR functionality useful will be more likely to accept the technology. However, we hypothesize that the relationship between PHR functionality and behavioral intentions is moderated by the communications the patient receives from the organization about the PHR. Therefore, we focused on the interactive effect of these factors.

### Organizational Factor

An important aspect of the environment that influences behavior is the information received through mass communication [43]. SCT is grounded in the notion that most external stimuli influence behavior through cognitive processes that determine which external events will be attended to, retained, and deemed important. Communication tactics reflect the extent to which an individual hears about the PHR through different channels such as email messages, posters, recorded phone messages, or providers. Organizations often design marketing messages to raise awareness of the benefits of the system to increase adoption of the technology. Patients who reported being exposed to more of these messages should be more aware of the benefits of the system. When this awareness of benefits is combined with the perception that the PHR functionality is of value, higher intentions to use the PHR should result.

*Hypothesis 3: Communication tactics will enhance the positive association between perceptions of the technology features and intentions to use the PHR tool.*

## Methods

### Data Collection Site

To test the research hypotheses, we collected data during the first 3 months post go-live of the deployment of the PHR with secure messaging tool implemented at Elmendorf Air Force Base in Alaska in December 2010. Approximately 26,000 individuals over the age of 18 were enrolled for receipt of health care at the Elmendorf military treatment facility (MTF) provided by a medical group staff of approximately 150. Initial goals associated with the PHR project included improving the quality of health care patients received, increasing staff productivity, decreasing staff workload, and enabling patients to have more control over their own health information. The tool was named “MiCare” to signal to patients that it would afford them greater control over their care.

### Procedure

Several weeks in advance of system go-live, patient registration cards were provided to the MTF. To register, patients visited the MTF and showed their military ID to the registration staff located at enrollment desks in the lobby. Once their information was entered into the system, the system automatically generated an email with a link to complete the registration process. Registered users’ data was extracted from existing Air Force medical databases to populate the PHR. Periodic updates kept the data current and consistent with the clinical “database of record”. Once the registration process was completed, the user could access the PHR tool from any Web-enabled computer (a screenshot of the Home tab is provided in Multimedia Appendix 1).

After initial registration, users received an email requesting their participation in an electronic survey to measure baseline expectations about the system and other individual characteristics. If the user chose not to participate in the survey at the initial request by selecting the “not now” option, 2 reminder emails were sent, one week apart. If the user agreed to complete the survey, the system assigned a unique identifier to the respondent to de-identify them for study purposes while also facilitating the matching of survey responses with existing data from military databases. We obtained patient demographics and health condition variables from existing Air Force databases to serve as control variables.

### Measurements

We used multi-item scales for all variables, relying on prior research for scales wherever possible. Because the data collection occurred prior to hands-on use of the tool, items were worded to reflect the respondent’s *expectations* about the use of the system (eg, for each of the features of the tool listed below, please indicate how useful you believe it *will* be for your personal health information management), formed on the basis of information they received about the tool. Drawing upon prior work in technology acceptance and use [44-45], the survey included a validated 3-item measure for future use intentions to measure the degree to which the patient planned to utilize the tool in the future, scored on a 7-point Likert scale, with anchors of “strongly disagree” (1), “neutral” (4), and “strongly agree” (7).

To gain a more granular understanding of the types of functionality that would be most valuable for patients, the survey included a list of 17 PHR features (eg, link to information about potential drug interactions, store and manage medical images, record and manage health care expenses) that respondents scored on a 1-7 Likert Scale anchored with “not at all useful” (1), “neutral” (4), and “very useful” (7). These features were selected based on the specific requirements that had been identified during the extensive requirements analysis performed by the research team and the software contractor who developed the PHR system for the Air Force. Requirements analysis included interviews with 20 patients and 3 patient focus groups. For tool empowerment potential, we developed a 5-item scale that tapped into the patients’ beliefs about whether the use of the tool would make the individual feel more empowered, more in control, more informed, better prepared, and more organized.

Baseline patient activation (the knowledge, skill, and confidence for self-management) was assessed using the 13-item patient activation scale from Hibbard et al [37] that has been validated across a number of studies. Respondents indicated their overall satisfaction with their provider using 3 items scored on a 7-point Likert scale. To evaluate the effectiveness and reach of the different communication tactics, we asked respondents how much they had heard about the PHR pilot through 9 different communication channels, including posters, website, base newspaper articles, and recorded phone messages.

Because computer skills have been previously linked to PHR adoption behavior [46], for control purposes, we asked respondents to rate their computer skills. Additional controls from the military databases included gender, age, sponsor pay grade, and the total number of chronic disease diagnoses such as asthma, hypertension, diabetes, etc, to serve as a proxy for general health. Finally, the survey contained an open-ended question asking users to provide any other comments or feedback they had.

Prior to conducting the full study, we did extensive pre-testing of the survey instrument to ensure that the scales were valid and reliable, and that respondents interpreted each question the way it was intended. The final survey contained a total of 81 items, together with 1 open-ended response. We performed cognitive testing with 6 subjects who completed the survey while 2 researchers were present, and provide feedback on the format and wording of the questions. This was followed by a field pre-test where we solicited patients in a military treatment facility that was different from the main study site. We obtained responses from 38 patients. Analysis of the pre-test data supported the validity and reliability of the measurement scales. We also confirmed that the survey could be completed by the respondent in less than 10 minutes.

## Results

### Descriptive Statistics of the Sample

**Figure 2:**
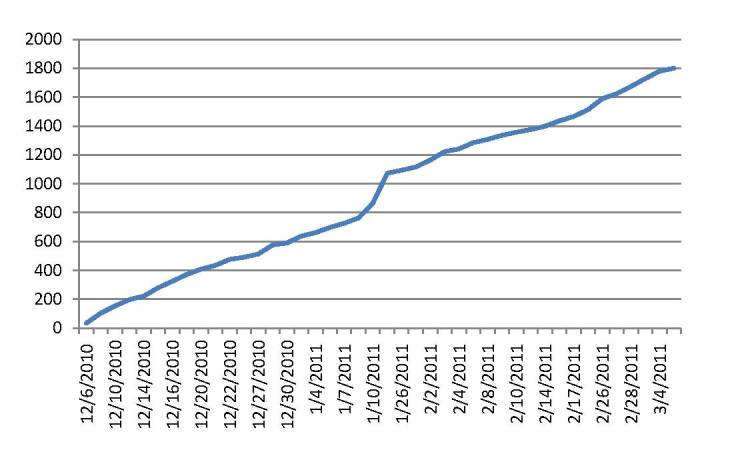
Baseline patient enrollment.

The adoption trajectory of the system over the first 3 months post go-live is shown in Figure 2. Over this time period, of the approximately 26,000 adult patients invited to register, 1801 completed the registration, yielding an adoption rate of 7%. We received 283 responses to the email survey requests, which represent a 16% response rate. Of the survey respondents, 64% (181/283) were female. Over half of the respondents rate their computer skills as quite extensive or very extensive.

Table 1 summarizes demographic information for: (1) the Elmendorf population, (2) the early adopters of the PHR, and (3) our survey sample. The survey sample was significantly older, higher paid, more sick, and more likely to be female than the Elmendorf population. The survey sample was also older, higher paid, more sick, and more likely to be active duty than early adopters who chose not to complete the survey. These differences were consistent with findings from other studies which examined usage patterns of secure messaging and found usage to be higher for women, individuals with higher levels of education, and increased morbidities [47,48]. Education information was not available from the Air Force databases, but income was often correlated with education [49]. Since registration for the PHR was done at the MTF upon presentation of a military ID, it is possible that individuals seeking treatment for an illness or older patients who required more clinic visits tended to enroll more than others simply because they visited the clinic. More dependents enrolled for the PHR, yet more active duty personnel responded to the survey requests. This may reflect a greater sense of duty or responsibility to participate in the research on the part of the active duty personnel. A comparison of mean scores on summated scales between early versus late responders to the survey revealed no significant differences [50].

**Table 1:** Demographic profile of early adopters.

Variable		Elmendorf Population (1) N=26,096	PHR Early Adopters (2) N=1518	Survey Respondents (3) N=283
**Demographics**				
	Gender, (Male=1, Female=0)	0.46	0.37, (1)^a^	0.36, (1)^a^
	Age, years	40.0	32.1, (1)^a^	47.2, (1)^a^,(2)^a^
	Sponsor pay grade, numeric scale 1-9	5.48	5.40, (1) ^a^	5.69, (1)^a^,(2)^a^
	Number of dependents, sum	0.72	0.63	0.60
	Dependents vs active duty, (Dependent = 1, active duty = 0)	0.52	0.64, (1)^a^	0.55, (2)^a^
**Medical Condition**				
	Average total chronic diseases, sum	0.39	0.49, (1)^a^	0.63, (1)^a^,(2)^a^

*This variable is significantly different from the same variable in columns (1) or (2), as labeled in the heading.

### Data Analysis

We first performed factor analysis to confirm the psychometric properties of the measurement scales. Principal components factor analysis of the 17 items used to assess the importance of various features of the PHR tool yielded a two-factor solution. The first factor consisted of 9 items related to the tool’s capability to store and track patient historical information, and therefore, we labeled it the “record keeping” feature of the tool. The second factor consisted of 8 items related to the tool’s potential to provide the patient “health care process management support” (eg, exchanging information between providers, reminders about appointments). All constructs and the corresponding items used for the statistical analysis are presented in Multimedia Appendix 2.

The patient activation items loaded on 2 factors that represent different stages of patient activation [36,37]. The first 7 items loaded together to form a factor representing a patient’s belief about their role in self-health management and their confidence and knowledge in their own ability to take action (PA-knowledge/beliefs). The last 6 items loaded on the second factor that captures a patient’s actual actions and ability to maintain appropriate self-health activity when under stress (PA-actions/maintenance). In addition, the 9 communication tactics deployed loaded on 2 factors. The first factor included 7 communication mechanisms that were impersonal in nature, (eg, posters, emails) while the second included 2 personal communication mechanisms (ie, registration desks, providers/staff). Table 2 shows the reliability (Cronbach alpha), means, and SDs for the variables and correlations between the constructs. Summated scales for all research constructs were used in the statistical analysis.

We estimated the research model using moderated multiple regression in SPSS. Intention to use was regressed on all the independent variables shown in Figure 1. We first entered the control variables into the regression, followed by the main effects. To model the hypothesized moderating relationships, we included 6 additional variables representing the product of PA-knowledge/beliefs and Perceived Tool Empowerment Potential, PA-actions/maintenance and Perceived Tool Empowerment Potential, Impersonal Communication Tactics with the 2 factors for Perceived Value of Tool Functionality, and Personal Communication Tactics with the 2 factors for Perceived Value of Tool Functionality. The regression equation included control variables for gender, age, sponsor pay grade, computer skill level, dependent status, and general health. A confidence level of 95% was utilized for the purposes of hypothesis testing. We used a listwise deletion procedure for missing data.

**Table 2:** Descriptive statistics: reliability, means, SDs, and correlations (N=283).

Construct	Reliability (# of items)	Mean (SD)	1	2	3	4	5	6	7	8	9
Intentions	.91(3)	5.98 (1.15)	1								
PA-knowledge/ beliefs	.90(7)	6.40 (0.71)	.48**	1							
PA-actions/ maintenance	.89(6)	5.62 (1.11)	.31**	.58**	1						
Provider satisfaction	.96(3)	6.05 (1.26)	.44**	.50**	.40**	1					
Tool empowerment potential	.95(5)	5.97 (1.06)	.67**	.48**	.29**	.40**	1				
Record keeping functions	.94(9)	6.26 (1.12)	.51**	.41**	.31**	.34**	.53**	1			
Health care process management support functions	.95(8)	6.29 (1.07)	.57**	.41**	.26**	.35**	.64**	.84**	1		
Communication tactics (impersonal)	.83(7)	2.10 (1.08)	-.03	-.01	.16*	-.05	.10	-.01	.04	1	
Communication tactics (personal)	.72(2)	3.03 (1.29)	.16*	.07	.10	.07	.24**	.11	.16*	.37**	1

* *P*<.05

** *P*<.01

**Figure 3:**
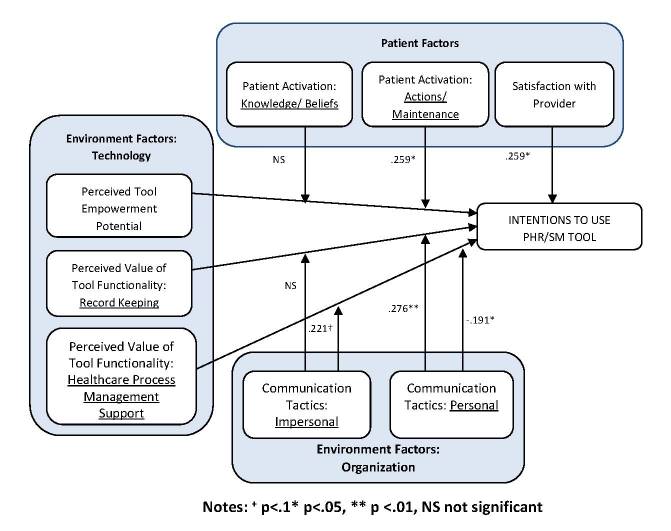
Results of model estimation.

Figure 3 summarizes the results of the regression analysis and depicts the significant predictors of use intentions for the PHR tool. Provider satisfaction was significantly associated with intentions to use. PA-actions/maintenance positively influenced the relationship between tool empowerment potential and intentions to use the tool, while PA-knowledge/beliefs did not. Intentions to use the tool were highest for patients indicating high levels of competence in managing their own health (PA-actions/maintenance) who also believed the tool was likely to make them feel empowered. PA-actions/maintenance had little effect on intentions for patients who did not believe the tool would make them feel empowered.

Both personal and impersonal communication tactics interacted with perceived value of the health care process management support features of the tool to increase use intentions. Intentions to use were highest for patients who perceived the health care process management support feature to be very useful and who also heard a lot about the tool through either personal or impersonal communication channels. Contrary to what was hypothesized, communication tactics that were more personal in nature had a negative interactive effect on the relationship between value of the record keeping function of the PHR and intentions to use. Hearing a lot about the tool through personal communications tended to decrease a patient’s intentions to use the tool when their perceptions of the usefulness of the record keeping functions of the tool were high. If the patient’s perceptions of the usefulness of the record keeping functions of the tool were low, hearing a lot about the tool through personal communication tactics had no influence on intentions.

**Figure 4:**
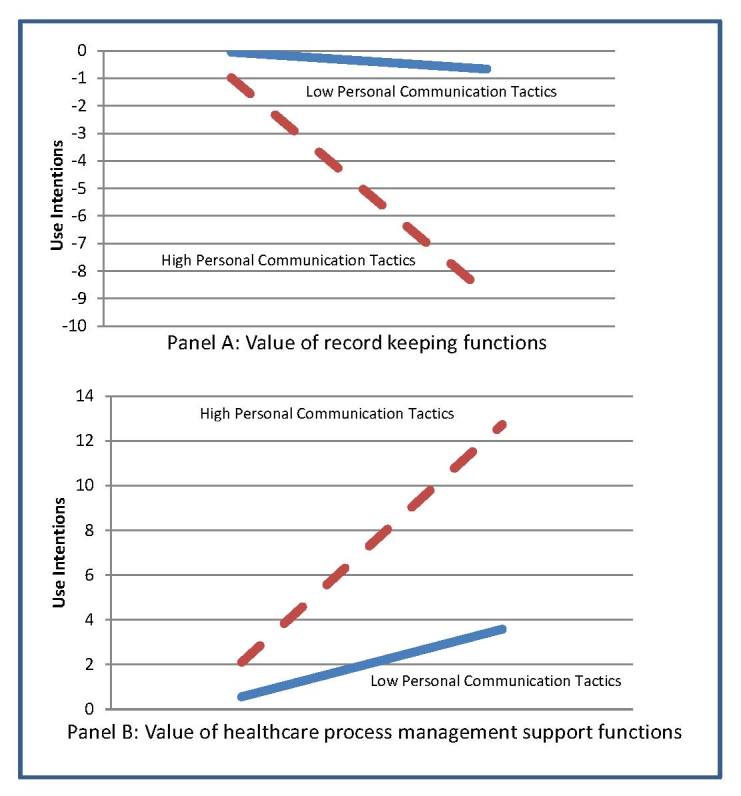
Interactions between perceived value of tool functionality and personal communication tactics.

Interaction effects for personal communication tactics and perceived value of the PHR tool on use intentions are depicted in Figure 4. Impersonal forms of communication had no influence on the relationship between the perceived usefulness of the record keeping functions of the PHR and intentions to use.

In summary, hypothesis 1, predicting a significant relationship between satisfaction with health care provider and intentions to use the PHR tool, was supported. The results also partially support hypothesis 2, which proposed a significant positive interaction between the perceived value of the PHR tool and patient activation in their effects on intentions to use (the interaction was significant for PA-actions/maintenance but not for PA-knowledge/beliefs). Finally, we found partial support for hypothesis 3, which proposed that communication tactics conditioned the effects of perceived value of record keeping and health care management process support functions on intentions to use. Collectively the hypothesized predictors explained 42% of the variance in behavioral intentions to use the PHR tool.

## Discussion

### Principal Findings and Implications

In this study, we used SCT as the basis for building and testing a model to predict patient acceptance of a PHR tool sponsored by the employer. Our findings supported a mutual and reciprocal relationship among the individual and environmental determinants of behavioral intentions to use the PHR. We found evidence that patients who were more satisfied with their providers were more likely to accept the PHR tool. In addition, perceptions of the 2 factors present in the environment, the technology, and organizational communication tactics, interacted to influence behavioral intentions. Finally, patient activation, reflecting the extent to which individuals felt confident in health self-management, interacted with perceptions of the tool’s ability to empower the individual, a technology environmental factor.

It is widely known that a strong patient/provider relationship can result in better patient outcomes [28-30], yet little is known about the effects of this relationship on consumer health IT acceptance. This study demonstrated that the reach of a strong connection extends to patient acceptance of new technologies as well. Although there has been limited studies to date, there are indications that operational and productivity gains may be realized with patient use of systems such as the one studied here [51-53]. More benefit can be gained by all members of the patient-provider system if providers can encourage patients to use the PHR systems more rapidly and extensively by fostering stronger relationships with them.

We found that use intentions were significantly affected by the perceived value of the various features offered in the PHR, but this relationship was contingent on the communication tactics deployed by the employer. For patients who did not perceive PHR functionality as valuable, communication efforts had no significant influence on intentions. However, for patients who perceived the health care process management support features of the tool to be of significant value, communication efforts served to increase their intentions to use the tool. This was true for both impersonal and personal forms of communication. Intriguingly, for patients who perceived the record keeping functions to be particularly important, personal communication had a negative influence on intentions to use the PHR while impersonal change management efforts had no influence on the relationship.

One possible explanation for the findings related to the communication tactics and the two types of functionality and intentions is in the specific capabilities and benefits stressed in the materials used by the implementation team throughout the project, that is, the content of the communication. Textbox 1 depicts these themes, the majority of which relate to exchanges between the health care system and the patient (what we label as health care process management support functions) and less about the patient’s personal record-keeping functions.

**Key Phr Capabilities and Benefits Used in Marketing Materials.**

MiCare allows you to take command of your health care:

request your next appointment
request medication renewals
receive your test and lab results
maintain a PHR to manage your health
communicate online with your health care team about non-urgent symptoms
avoid unnecessary office visits and telephone calls
request a copy of your immunization record
access a large library of patient education materials

Because the content of communication materials focused on health care process management support functions, it may be that the record-keeping functionality available within the tool was inadvertently downplayed. Perhaps, in personal exchanges with providers and staff or at registration desks (ie, personal communication), the emphasis may have been even more on the health care process management functions. As a result, patients may have walked away from these interactions with the impression that record keeping functions were minimally provided in the tool or not at all provided. For patients who perceive functionality to be very useful, if they were given the impression it was not available in the tool during these personal communications, it would likely lower their intentions to use it. An important implication of this finding is that communications from providers, staff, and other volunteers working at information/registration desks must convey balanced messages about the functionality of the tool.

We also found evidence of a positive interaction between the tool’s empowerment potential and patient activation on intentions to use the PHR. Patients who indicated the tool would make them feel more organized and in control of their health information demonstrated higher intentions to use, which was enhanced for highly activated patients. Thus, patients who were beginning to take actions to manage their own health and felt confident they could maintain these activities going forward were more likely to use the tool when they believed it would further enhance their capabilities to self-manage their health condition.

### Limitations

Our study has some limitations that also represent useful opportunities for future work. First, we examined overall provider satisfaction. Future research could investigate patient satisfaction with their provider at a more granular level (eg, competence, thoroughness, respectful attitude, active listening skills, responsiveness to questions) to determine if specific dimensions of provider satisfaction influence technology acceptance [54]. Second, we examined intentions and not actual behavior. However, a robust body of evidence demonstrated that intentions predict behavior [29-31], thereby mitigating this concern. Future work should attempt to measure actual use of the PHR. Third, the sample only included those who registered for the system, that is, the early adopters. While the sampling procedure allowed us to determine whether the hypothesized factors explain variance in behavioral intentions for this population, surveying those who did not register for the system would provide useful insight into factors driving non-adoption. Although the hypothesized model explains substantial variance in intentions to use, a related opportunity for future work is to extend the model to include additional organizational factors such as management support and training that have been shown in prior work to predict use intentions [24]. Fourth, a survey response rate of 16% (283/1801) resulted in a sample that was large enough to test the proposed research model. However, results must be interpreted keeping the possibility of response bias in mind. Demographics of the survey respondents are consistent with other work that has examined the usage of other health information technologies by patients. Fifth, although we had a detailed list of all communication tactics employed during the rollout, we studied the content of communication at a high-level, focusing only on the themes used in the communication material. Future research could conduct more detailed qualitative analyses of how employees respond to different communication channels and content, and the quality of the channels to better understand what type of messages are more likely to promote greater use intentions. Finally, with respect to generalizability of findings, the hypothesized relationships are derived from a strong evidence base of theory and prior empirical work. Thus, although the study needs to be replicated across different types of organizations before broader generalizations can be made, we expect the findings to extend to contexts that are similar in that the employer is providing the PHR as a service to the employee and usage of the system is volitional rather than mandated.

### Conclusions

Despite significant policy interest in promoting patient empowerment and the use of consumer health IT and mounting evidence suggesting that PHR use can reduce medical errors [55] and improve the patient-provider relationship [56] among other positive outcomes, the adoption and use of PHRs by consumers has been disturbingly slow [12]. Success of PHR technology may well lie in sponsorship by an organization such as an employer, insurer, or provider. This study is among the first to provide insight into factors that an organization could leverage to increase acceptance of a sponsored PHR.

Our study reinforces findings in other areas of health, which stress the importance of an involved patient. Just as it is less realistic to expect a hypertensive patient to consistently test blood pressure levels at home, exercise to lose weight, and follow other health-management behaviors in the absence of understanding about the health condition or a lack of confidence in his/her capability of self-management [37], it is less realistic to expect a similar patient to accept and consistently use a PHR. Hibbard et al [38] found that patient activation could be changed and that improvement in activation levels resulted in improved self-management behaviors. Providers who were able to improve patient activation levels may deliver more effective and efficient care [38]. PHR acceptance may improve to the same extent as providers are willing to motivate and increase the levels of patient activation through a variety of different interventions (eg, seminars, disease managers, counseling). Further underscoring the importance of the provider’s role in the process is the finding suggesting the positive effect of a satisfactory patient-provider relationship in PHR acceptance. Finally, we demonstrated that it is not sufficient for potential adopters to find PHR functionality useful, as the main effect of perceived usefulness was not significant in predicting use intentions. High intentions to use the PHR were created by a combination of patients’ perceived usefulness and the communication tactics used during system rollout. This suggests that communication from the employer on the capabilities and benefits of the system sends a powerful advocating message to the individuals that, when combined with their own perceptions of the value of the system, translates into high intentions to use the system. The health care process management support function of the PHR represents a two-way street between the patient and provider (eg, scheduling appointments, exchanging messages). It is not surprising that the patient needs to perceive that the other party in the exchange believes in the benefits of the system as well. Communication tactics help reinforce this message. Organizations planning a PHR implementation should carefully craft a communication strategy suited to their organization’s needs to improve the likelihood of a high adoption rate, resulting in the highest return on their investment in the technology.

## Multimedia Appendix 1

PHR/SM home tab.

## Multimedia Appendix 2

Measures used in study.

## Abbreviations

AFMS: Air Force Medical Service
MTF: military treatment facility
PHR: personal health record
SCT: social cognitive theory
SM: secure patient-provider messaging
