# Supplementary figures and images for: If We Offer it, Will They Accept? Factors Affecting Patient Use Intentions of Personal Health Records and Secure Messaging
Source: J Med Internet Res. 2013 Feb 26;15(2):e43. doi: 10.2196/jmir.2243 (PMC3636193; doi:10.2196/jmir.2243)

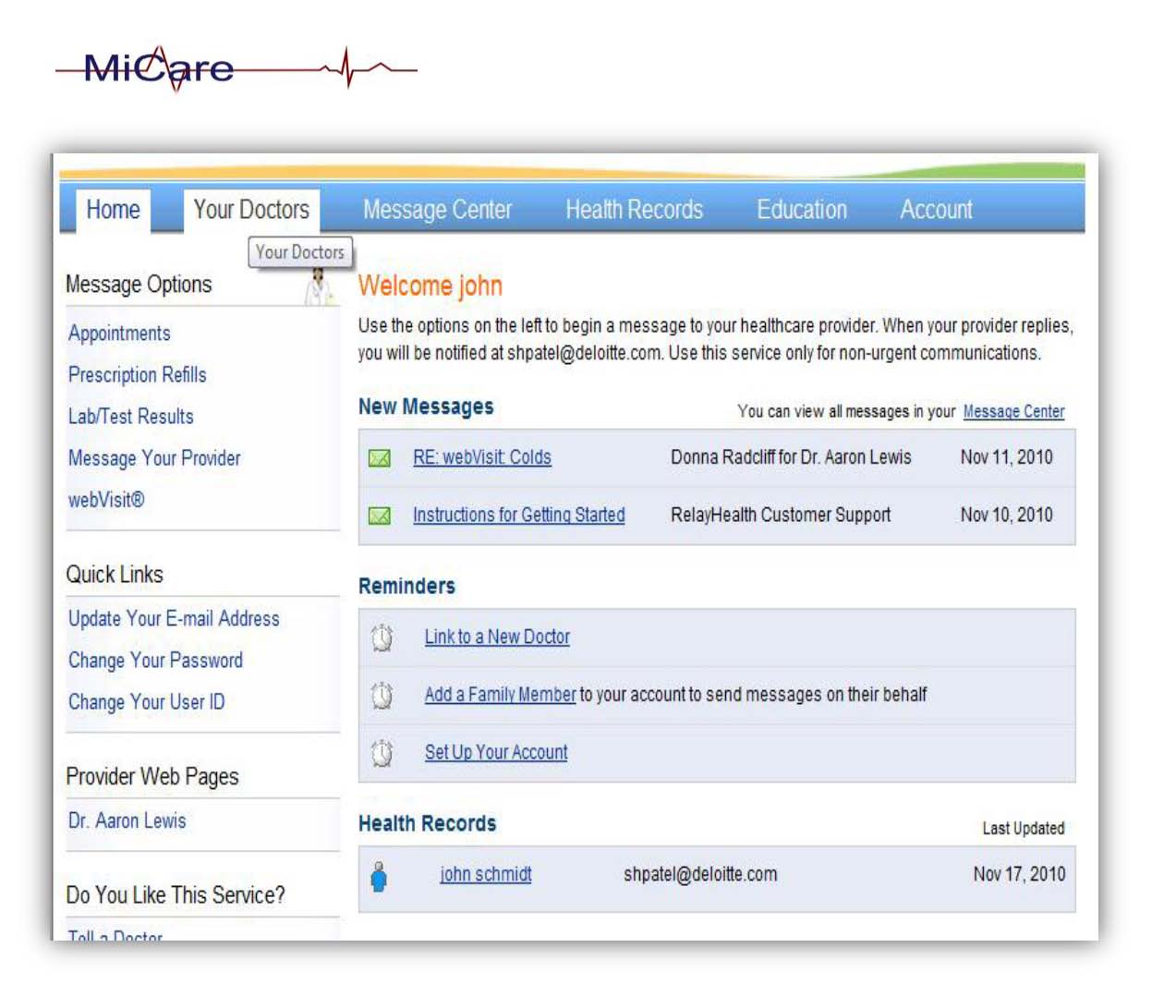

Supplement: Supplementary file 1 [file jmir_v15i2e43_app1.jpg]
