# Supplementary material for: If We Offer it, Will They Accept? Factors Affecting Patient Use Intentions of Personal Health Records and Secure Messaging
Source: J Med Internet Res. 2013 Feb 26;15(2):e43. doi: 10.2196/jmir.2243 (PMC3636193; doi:10.2196/jmir.2243)
Supplement: Supplementary file 2 [file jmir_v15i2e43_app2.pdf]

Construct and definition	Items
Intentions	Please indicate your level of agreement with the following statements by placing an X in the appropriate column (1: Strongly disagree– 7: Strongly agree)
Intentions to use the PHR in the future	• I intend to use MiCare in the near term<br>• I believe my use of MiCare will be more extensive in the future<br>• I intend to use MiCare more frequently in the future
Patient Activation	Please indicate your level of agreement with the following statements by placing an X in the appropriate column (1: Strongly disagree–7: Strongly agree)
<u>Knowledge/Beliefs:</u><br>Beliefs about role in managing their own health and their confidence and knowledge in their own ability to take action	• When all is said and done, I am the person who is responsible for managing my health condition<br>• Taking an active role in my own health care is the most important factor in determining my health and ability to function<br>• I am confident that I can take actions that will help prevent or minimize some symptoms or problems associated with my health condition<br>• I know what each of my prescribed medications do<br>• I am confident that I can tell when I need to go get medical care and when I can handle a health problem myself<br>• I am confident I can tell my health care provider concerns I have even when he or she does not ask<br>• I am confident that I can follow through on medical treatments I need to do at home
Patient Activation	Please indicate your level of agreement with the following statements by placing an X in the appropriate column (1: Strongly disagree–7: Strongly agree)
<u>Actions/Maintenance:</u><br>Beliefs about actual actions and ability to maintain appropriate	• I understand the nature and causes of my health condition (s)<br>• I know the different medical treatment options available for my

health self-management activity under stress	health condition<br>• I have been able to maintain the lifestyle changes for my health that I have made<br>• I know how to prevent further problems with my health condition<br>• I am confident I can figure out solutions when new situations or problems arise with my health condition<br>• I am confident that I can maintain lifestyle changes like diet and exercise even during times of stress
Provider Satisfaction	Please indicate your level of agreement with the following statements by placing an X in the appropriate column. (1: Strongly disagree–7: Strongly agree)
Patient satisfaction with the relationship with the provider	• Overall, I am satisfied with my provider<br>• My provider meets my needs<br>• My interactions with my provider are positive
Tool Empowerment Potential	Please think about how the use of the MiCare tool makes you feel and indicate your response below by circling the appropriate number.
Perceptions about the extent to which use of the PHR would empower the individual	• Less Empowered (1) – More Empowered (7)<br>• Less In Control (1) – More In Control (7)<br>• Less Informed (1) – More Organized (7)<br>• Less Prepared (1) – More Prepared (7)<br>• Less Organized (1) – More Organized (7)

Value of Tool Functionality<br><br>Record Keeping Functions: Perceptions about the usefulness of various record keeping features of the PHR	For each of the features of the MiCare PHR/SM tool listed below, please indicate how useful you believe it will be for your personal health information management and health care activities by selecting the appropriate option. (1: Not at all useful – 7: Very useful)<br>• Track lab results over time<br>• Track vital statistics (eg, blood pressure, weight)<br>• Record my medications<br>• Record my immunizations<br>• Record my medical procedures<br>• Record my surgeries<br>• Track my doctor visits<br>• Record my personal medical history<br>• Record my family's medical histories
Value of Tool Functionality<br><br>Healthcare Process Management Support Functions:<br><br>Perceptions about the usefulness of process management features of the PHR	For each of the features of the MiCare PHR/SM tool listed below, please indicate how useful you believe it will be for your personal health information management and health care activities by selecting the appropriate option. (1: Not at all useful–7: Very useful)<br>• Store and manage medical data from a variety of health care provider sources (eg, data I type in myself or images obtained from tests conducted by non-military health care providers)<br>• Assurance that my medical information is private and secure<br>• Ability to securely message my health care providers<br>• Ability to store medical data online<br>• Ability to have medical information available anytime from any web-enabled computer<br>• Ability to print and/or exchange medical information to share with my health care providers

	• Health-specific reminders (such as check-ups, tests, etc.)<br>• Having my emergency information immediately accessible (eg, blood type, allergies, emergency contact)
Communications Tactics<br><br><u>Impersonal:</u><br><br>The use of impersonal (mass media) communication channels for informing individuals about the PHR	How much have you heard about the MiCare PHR/SM pilot project via the following mechanisms? (1: Not at all–7: A lot)<br>• Posters<br>• Website<br>• Emails<br>• Flyers/Reminder cards<br>• Base newspaper articles<br>• Recorded phone messages<br>• Briefings/Live demonstrations
Communication Tactics<br><br><u>Personal:</u><br><br>The use of personal communication channels for informing individuals about the PHR	How much have you heard about the MiCare PHR/SM pilot project via the following mechanisms? (1: Not at all – 7: A lot)<br>• Enrollment/Registration Desks<br>• Provider/Clinic Staff
